# Towards Intelligent Zone-Based Content Pre-Caching Approach in VANET for Congestion Control

**DOI:** 10.3390/s22239157

**Published:** 2022-11-25

**Authors:** Khola Nazar, Yousaf Saeed, Abid Ali, Abeer D. Algarni, Naglaa F. Soliman, Abdelhamied A. Ateya, Mohammed Saleh Ali Muthanna, Faisal Jamil

**Affiliations:** 1 Department of ITT, The University of Haripur, Haripur 22620, Pakistan; 2 Department of Computer Science, University of Engineering and Technology, Taxila 47050, Pakistan; 3 Department of Computer Science, Government Akhtar Nawaz Khan (Shaheed) Degree College KTS, Haripur 22620, Pakistan; 4 Department of Information Technology, College of Computer and Information Sciences, Princess Nourah bint Abdulrahman University, P.O. Box 84428, Riyadh 11671, Saudi Arabia; 5 Department of Electronics and Communications Engineering, Zagazig University, Zagazig 44519, Egypt; 6 Institute of Computer Technologies and Information Security, Southern Federal University, 347922 Taganrog, Russia; 7 Department of ICT and Natural Sciences, Faculty of Information Technology and Electrical Engineering, Norwegian University of Science and Technology (N.T.N.U.), Larsgårdsvegen 2, 6009 Ålesund, Norway

**Keywords:** machine learning, content pre-caching, zone, congestion control, VANET

## Abstract

In vehicular ad hoc networks (VANETs), content pre-caching is a significant technology that improves network performance and lowers network response delay. VANET faces network congestion when multiple requests for the same content are generated. Location-based dependency requirements make the system more congested. Content pre-caching is an existing challenge in VANET; pre-caching involves the content’s early delivery to the requested vehicles to avoid network delays and control network congestion. Early content prediction saves vehicles from accidents and road disasters in urban environments. Periodic data dissemination without considering the state of the road and surrounding vehicles are considered in this research. The content available at a specified time poses considerable challenges in VANET for content delivery. To address these challenges, we propose a machine learning-based, zonal/context-aware-equipped content pre-caching strategy in this research. The proposed model improves content placement and content management in the pre-caching mode for VANET. Content caching is achieved through machine learning, which significantly improves content prediction by pre-caching the content early to the desired vehicles that are part of the zone. In this paper, three algorithms are presented, the first is zone selection using the customized algorithm, the second is the content dissemination algorithm, and the third is the content pre-caching decision algorithm using supervised machine learning that improves the early content prediction accuracy by 99.6%. The cache hit ratio for the proposed technique improves by 13% from the previous techniques. The prediction accuracy of the proposed technique is compared with CCMP, MLCP, and PCZS+PCNS on the number of vehicles from 10 to 150, with an improved average of 16%. Finally, the average delay reduces over time compared with the state-of-the-art techniques of RPSS, MLCP, CCMP, and PCZS+PCNS. Finally, the average delay shows that the proposed method effectively reduces the delay when the number of nodes increases. The proposed solution improves the content delivery request while comparing it with existing techniques. The results show improved pre-caching in VANET to avoid network congestion.

## 1. Introduction

The term vehicular ad hoc networks (VANET) is adapted from the ad hoc nature of the networks. VANET is an emerging new technology to integrate the capabilities of new-generation wireless networks into vehicles. The idea of VANET is to provide: (1) ubiquitous connectivity while on the road to mobile users who are otherwise connected to the outside world through other networks at home or the workplace, and (2) efficient vehicle-to-vehicle communications that enable intelligent transportation systems (ITS). Therefore, vehicular ad hoc networks are also called intervehicle communications (IVC) or vehicle-to-vehicle (V2V) communications [1].

An intelligent transportation system (ITS) uses information and communication technologies (ICT) to connect, communicate, and share information more efficiently, smarter, and in greater quantity. Vehicular ad hoc networks (VANET) provide wireless communication for efficient and remote communication among vehicles in transportation services. In VANET, the vehicles communicate using wireless communication among other vehicles, roadside units (RSUs), and base stations (BSS). Safety messages and information exchange are among the most important aspects of modern communication in VANET [2]. The messages were exchanged in VANET to save passengers and drivers from road accidents, save many lives, and handle traffic congestion. Communication is only possible and provides an effective way to compensate differently. In the last few years, the data and their exchange among multiple devices are still issues that enhance the problem of data management and data establishment policies. Especially in the last decade, data production has been enhanced unprecedentedly. According to tech joint CISCO, data management, and exploration were enhanced up to 7.2 exabytes per month in 2021 [3]. Mobile cloud technologies enhanced data requests and responses, which established the functioning of multiple mobiles and other cloud servers. There are several reasons which come up in the form of vehicular ad hoc networks (VANET), mobile ad hoc networks (MANET), cloud ad hoc networks (CANET), etc. These no longer support central command servers with application request and response requirements [4]. ITS offers communication among multi-hop wireless communication with aspects to increase the number of ranges established for the communication aspects of the live transmission power and enhance the working of different scenarios. The exchange of information in this network is possible by exchanging multiple wireless communication networks that establish the network range and enhance the trustable and wireless communication protocols’ working mechanism [5]. 

**Figure 1:**
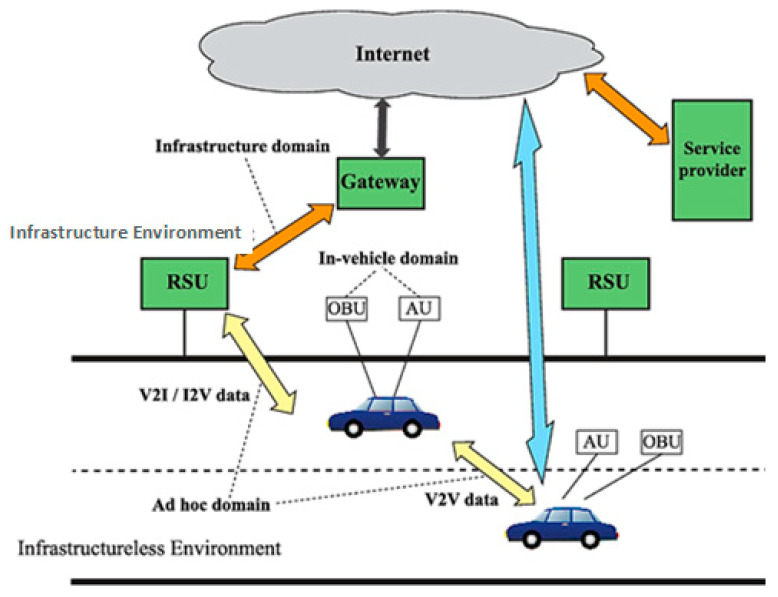
VANET structure for AU, OBU, and RSU [2].

Long-distance communication in VANET increases vehicle load, low quality of experience (QoE), and long transmission delay. These may cause network congestion and provide robust and effective road safety and condition overloading in different network ranges [6]. On the other hand, centralized group management policies provide data privacy and security. The content request and response from the RSU or BSS may cause transmission delay and provide a range for the transmission equipment transmission parameters [7]. The delay may only be overcome through content pre-caching strategies implemented in VANET to support the quality of service, increase transmission time, the less average delay in content access and content requests, and content prediction accuracy inside the network to distribute and provide an effective mechanism to support and enhance the capital working of different parameters, and ultimately cache hit ratio for the different platforms. VANET provides three main components, which include: the application unit (AU), on-board unit (OBU), and roadside unit (RSU) [8]. Figure 1 provides the effective communication range for VANET architecture.

The dynamic connectivity and the short period of communication of the VANET cause very limited service coverage for vehicles and drivers to access the content. As every moving environment causes frequent service disruption, other related parameters cause the delay of the service and provide a significant enhancement for the dynamic coverage area. The delay in the content request may also cause server performance degradation. Pre-caching is one of the main solutions that caches the valuable content after pre-caching and enhances the working of wireless sensors to control and provide effective parametric values. The content request and response process may enhance the complete working functionality of the provided content request and response parameters. Handling all the contents and requests may cause service delays and content handling efficiencies [9].

The data produced by urban vehicles continue in nature, so predictions about road safety and traffic control are difficult. The speeds of an urban area’s vehicles are fast, so pre-time prediction of road conditions and accidents can save more disasters. Thus, designing an efficient data pre-cache prediction is one of the more efficient techniques to resolve these issues. The proposed protocols inform vehicles about interesting safety events and efficiently overcome disasters by early prediction of the results.

In VANET, content caching consists of different types. These types are not according to the content storage but the provided mechanism. The content caching stores the content on RSU, Cluster Head, or directly on the vehicular node. There are some techniques in which the content is stored sequentially. These techniques are presented only to download the cached content sequentially.

On the other hand, some techniques require content on-demand bases. These techniques efficiently place the content on demand whenever and wherever a vehicular node requires content. The BS, CH, or RSU cache the content on demand based. The last type of caching is content pre-caching approaches. These are more efficient than others because they predict early events from the content vehicles and provide an effective and collaborative mechanism for content pre-caching techniques [10]. There are still some types of techniques that are most relevant to enhancing the proposed techniques. Content pre-caching is the most efficient technique with respect to all other techniques to provide effective and reliable security and other details to provide the most efficient mechanism to support effectiveness. Adapting automatic behavior and working are significant achievements for VANET communication like machine learning.

**Figure 2:**
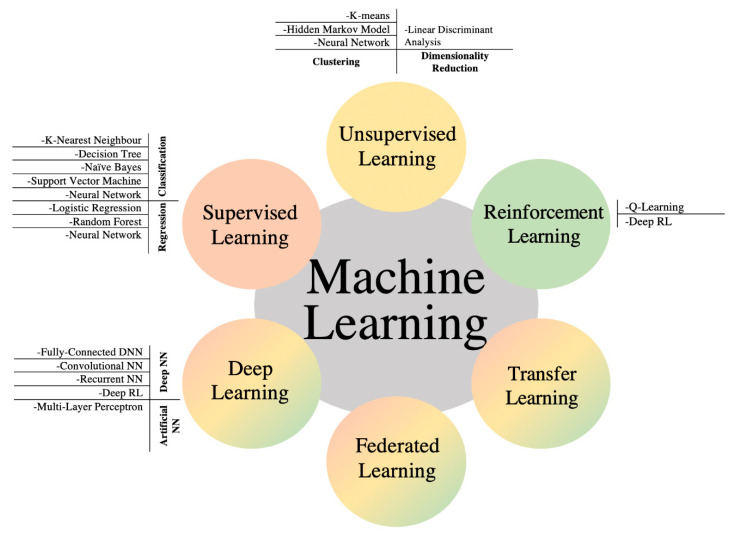
Machine learning models for VANET [11].

Machine learning is an attractive and emerging technique used for multiple application domains. An important technique is the VANET, wherein machine learning-based techniques are helpful in addressing different problem domains and discussing the relevant application scenarios. Nowadays, ML is applied in almost every area of VANET, like security, caching, training, routing, congestion handling, etc. Specially customized datasets are required to promote machine learning adoption in VANET. Figure 2 shows the ML models and algorithms. A typical model for the traditional ML consists of three phases: (1) the training phase, which takes raw data and pre-processes it to extract the features. The features are input into the ML model to learn patterns and classes of the data; (2) the testing phase, where the ML model tests a new set of data for classification based on its learning experience from the training phase; (3) the prediction phase, also known as the evaluation phase, where the working efficiency of an ML model is evaluated based on quality metrics (such as accuracy, false-positives, false-negatives, and so on).

### 1.1. Importance of the Research

The data produced by urban vehicles continue in nature, which predicts road safety and traffic control. It is a challenging process to predict [12]. The speeds of the urban area’s vehicles are fast, so pre-time predicts road conditions and accidents. If this happens, it can save us from disasters [13]. Thus, designing an efficient data pre-cache prediction is one of the more efficient techniques to resolve cache problems in VANET. The proposed methodology informs vehicles about interesting safety events, such as pre-decision in disastrous situations, and efficiently overcomes disasters through early prediction results [14].

In this paper, the Geocast-based technique is the most efficient data dissemination approach for VANET applications, especially for safety applications, because “security events only concern vehicles in the area close to the event’s location”. Moreover, this paper improves the prediction results for early road safety conditions through urban VANET based on machine learning. We improved the cache hit ratio, prediction accuracy, throughput, and average delay. The main idea is to secure applications to improve drivers’ and passengers’ road safety. Urban vehicle safety is one of the main reasons for the early prediction of road and accident conditions. These applications are based on data dissemination which is periodic or not enabling the state of the road and surrounding vehicles. The main benefit of this prediction is securing road conditions and other related operations in urban areas. The main idea is to deliver data about prediction with secure road conditions. In Geocast, a data dissemination message is sent only to the vehicle inside a specific geographical area called a zone of relevance (ZoR). This study handles the content pre-caching using machine learning under the secure parameter of blockchain technology to enhance network content throughput, minimize average delay, and minimize the hope count.

### 1.2. Problem Description

The idea is to secure and improve driver and passenger safety on roads. Urban vehicle safety is an idea to predict the road and accident conditions before any accident. The scenario is based on data dissemination which is periodic or not enabling the state of the road and surrounding vehicles. The continuity of the dynamic connections, limited coverage, fast vehicular movement, connection handover, and service interruptions lead to VANET content sharing degradation and high delay in content sharing. So, we focused on delay-sensitive services in VANET. It is better for service delivery to pre-cache the content when vehicles approach zones. The short time connecting vehicles to zones shows a delay in the content response process. The novelty of benefiting from early content prediction is enhancing road safety in urban areas.

The problem lies when content pre-aching is manual in recent research, which causes a delay in the network performance. In Geocast, a data dissemination message is sent only to the vehicle inside a specific geographical area called a zone of relevance (ZoR). The proposed mechanism disseminates safety messages in V2V and V2I scenarios. It is specially designed and suitable for the urban environment. The main goal of Geocast communication is to perform a safe information dissemination process within the zone of relevance (ZoR) and without the zone of forwarding (ZoF). This work defines the new secure pre-caching strategy to handle urban vehicle road and incident conditions through zone-based techniques. With high accuracy, the machine learning algorithm is used for early content prediction of road and traffic incidents.

In this paper, we have considered the below-mentioned questions during the task scheduling.

(i). How can a machine learning algorithm predict the pre-caching strategy to control traffic congestion?
(ii). How can congestion be controlled in VANET?
(iii). How to handle the pre-caching through the manager node from every zone?

### 1.3. Research Contributions

The main contribution of the proposed system is to improve road safety and transportation efficiency with wireless communication technologies as follows:

(i). The proposed technique disseminates safety messages using content pre-caching in V2V and V2I scenarios which saves the lives of passengers and drivers;
(ii). The proposed Geocast communication contributes to a safe information dissemination process within the zone of relevance (ZoR) and without the zone of forwarding (ZoF);
(iii). The machine learning technology provides early prediction of the content pre-caching with higher accuracy of 99.6% using zone base prediction to overcome further road disasters;
(iv). We design zone-based V2V and V2I communication using RSU, Interzone, and Intrazone vehicles, which enhance the content pre-caching availability and expand their coverage to a bigger zone. It is conducive to overcoming the network overhead and improves the cache hit ratio;
(v). The proposed VANET content pre-caching technique improves early prediction to reduce the network overhead;
(vi). We design and propose a pre-caching algorithm using machine learning. The tables and road topology information are recorded in the central manager node, and the proposed technique predicts the vehicle pre-caching early requests with high accuracy, and further content pre-caching is achieved using zone time and delay, which provision future requests in a timely manner to improve the reliability of pre-caching strategy and dynamic adaptability;
(vii). The performance of the proposed technique is compared with four previous techniques, and the machine learning algorithm is used to train the model for auto-prediction of content pre-caching, which shows high prediction accuracy. The results are evaluated by comparing the results with zone-based content pre-caching [12] and Geo-SID [13] using NS-2 over cache hit ratio, prediction accuracy, and average delay with time and number of vehicles. The proposed technique performs better than the previous pre-caching schemes in VANETs.

The rest of the paper is organized as follows. Section 2 presents the related work on content caching and machine learning-based models to improve the caching in VANET. Then, in Section 3, we outline our approach for the research and propose solutions to the relevant problems encountered in Section 1 of the paper. Then, Section 4 presents the proposed method with simulations using a content pre-caching approach. Section 5 concludes our proposed system, highlighting that our technique is straightforward for fault tolerance methodology.

## 2. Literature Review

The recent research is conducted based on the existing research methodologies and provides a thorough approach to research contributions. Zone-based content pre-downloading and caching strategies are implemented in the existing literature. The existing techniques use content pre-caching but lead us to a problem of real-time pre-caching decision-making. Multiple pre-caching strategies in VANET are implemented and discussed in this section. The content is cached based on popularity, node coordination, emergency condition, eviction policies, and centrality.

Additionally, we study the zone base selection and coordination for the content placement and caching requests. All these requests and parameter responses are studied and provided comprehensively to support and enhance efficiency. All the strategies presented in the literature show tradeoff between cache hit ratio, prediction accuracy, average delay, and node density in the proposed technique, which provides an efficient caching mechanism. 

Several strategies are implemented to target the hop count and face challenges during pre-caching content. These include a new technique in content pre-caching in VANET, an ICN-based VANET approach for name-based content caching, a game-based caching incentive scheme, a CCN-based caching in VANET environment, and pre-caching in a VANET environment with 5G communication mechanism. In addition, infotainment applications are considered to achieve the accuracy of the messages and provide the high-level application domain through edge computing, caching on RSU, and pre-caching on vehicles. The pre-caching chunks mechanism is adopted to achieve high network distribution and performance accuracy [15].

Ref. [16] proposed a new technique in content pre-caching in VANET. The authors in this approach use two algorithms first to select the zones through the pre-caching zone selection technique, and the second is the pre-caching node selection algorithm. Two PNZS and PCNS techniques are initiated for better and more effective knowledge discovery. Both algorithms collectively decide the pre-caching using a zone base system [17]. Authors use the ICN-based VANET approach for name-based content caching. The authors proposed in-network caching strategies to implement the proposed technique for efficient results. The contributions of the authors are to achieve lower cost and energy contributions. Still, on the other hand, the authors do not achieve the network throughput because of the caching of the content on respective RSUs. In ref. [18] the authors introduce a game-based caching incentive scheme. This approach works for the safety and security of VANET passengers and drivers. In ref. [19], the authors use a CNN-based VANET environment. The authors performed three main functions in this methodology. The first is to adopt the resource detection algorithm, and the second is to perform minimum vertex cover. The third one opposes collaborative caching based on socialized relations (CCBSR) to determine the caching point to solve the disconnect caused by the mobility of vehicles. 

In ref. [20], the authors introduce the VANET environment with a 5G communication mechanism. SDN and 5G are two promising technologies for VANET, which fulfilled the demand for bandwidth-sensitive applications and increased traffic data. The authors propose small base stations (SBS) that communicate and store the content on vehicles in V2V communication. The research aims to reduce network congestion and effectively place the content on targeted vehicles. In ref. [21], the author divides the content caching problem into three sub-problems. Infotainment applications are considered to achieve the accuracy of the messages and provide the high-level application domain through edge computing. A two-layer distributed content caching scheme is proposed to formulate the efficiency of the results. In ref. [22], the authors proposed caching on RSU and pre-caching on vehicles. Authors consider V2V and V2I communication for RSU caching and vehicular pre-caching. Content delivery time (CDT) is considered a novel technique to improve CDT in highly congested areas for traffic congestion and load balancing. In ref. [23], the authors worked on vehicular content-centric networks. User volubility is established to achieve the network considerably.

The pre-caching chunks mechanism is adopted to achieve high network distribution and performance accuracy. The author proposes a mechanism for pre-caching chunks of large content objects, such as videos, among RSUs. First, they adopt the hidden Markov model (HMM) to predict a user’s moving trajectory. Based on the time gap from the current location to the predicted location of the mobile user, the corresponding RSU can pre-cache the required video chunks and provide them to the user as soon as he arrives at the predicted location. Another approach mentioned in ref. [24] supports the hybrid opinion dynamics comprising averager, copier, and voter agents, which ramble as random walkers on a spatial network. In ref. [25], the authors present a machine learning-based content pre-caching scheme for V2V and V2I communication. The situation is limited to cache enhancement and cache control which shows that cache content is one of the techniques which places the content toward cache enhancement. The joint strategy is implemented using machine learning-based deep reinforcement learning to joint allocation strategy to minimize the weighted average delay of data acquisition. Edge-based content caching is implemented with reinforcement learning to enhance the knowledge related to the proposed caching framework. In ref. [26], the authors present realistic time-varying channels for the VANET approach. The Markov-based channels are common and provide effective, realistic collaboration among different techniques to get presented. A machine learning-based deep reinforcement learning approach is applied, which uses the deep network to simulate and provide effective information learning mechanism coordination. In ref. [27], the authors present joint caching, communication, and computational design problem in one study. The authors worked on the cost efficiency and excellence of the network to promote the caching strategies. 

In ref. [28], the authors presented the deep reinforcement learning-based content caching framework to present the knowledge and provide the computational capabilities to compute where to place the content based on the existing methodologies. The authors presented a new internet of connected vehicles methodology for the proposed technique to address vehicles’ challenges in accessing the appropriate contents. In ref. [29], the authors consider the optimal caching strategy to implement the proposed technique in this study. The personal information enabled the placement and caching enhancement approaches for vehicular ad hoc networks. Deep reinforcement learning is another technique implemented to resolve the current issues and challenges faced during the presented methodology for problem description and enhancement. High dimensional and time-variant features are implemented through the framework to contribute to and provide high-accuracy network resources. The authors integrate DRL and permissioned blockchain into vehicular networks for intelligent and secure content caching. They first propose a blockchain-based caching framework to enhance the content and promote the network features for effective content placement on target devices.

The zone-based content caching approach in VANET for congestion control using a machine learning framework has not been suggested to the best of our knowledge. This research proposes a framework for content pre-caching task execution at a very low cost. Furthermore, through the proposed framework, we ensure the content pre-caching for VANET vehicular devices over highway scenarios. To extensively motivate and introduce the proposed method the common limitations of the related works are presented in Table 1. Table 1 describes the common points and differences among previous work and how we resolve these issues in the proposed work. 

## 3. Materials and Methods

### 3.1. Proposed Zone-Based VANET Congestion Control Architecture Using ML

**Figure 3:**
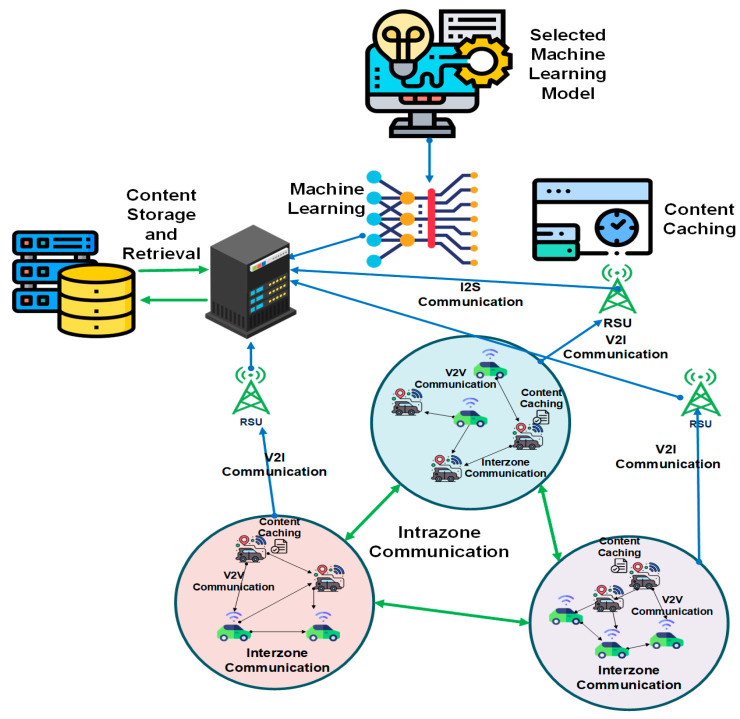
Zone-based content caching technique using machine learning.

This section briefly describes the research parameters and explains our proposed system with pre-caching in VANET through a machine learning technique. Figure 3 shows proposed model for Zone-based congestion control architecture. Initially, we discuss the construction of the zones through the clustering technique with parameters to select vehicles and define the threshold area with defined properties for individual zone. Roadside units (RSUs) and base station/content manager are added to collect the data through the zone-wise allocation of the vehicular adaptation [30]. This means pre-cached content initially fetched from RSUs. If an RSU cannot provide the content, then the request is forwarded to the base station/central manager through the RSU. Each zone is responsible for collecting RSUs. After that, we adopt machine learning to train the system to collect only urban vehicles. The data are collected through zones/clusters and forwarded to a trained machine learning model to predict cache hit ratio throughput average delay in putting the cached content through the machine learning mechanism. The intelligent CNN-based machine learning model predicts that only sensitive and safe messages handle content caching mechanisms. In this scenario, we consider both vehicle-to-vehicle (V2V) and vehicle-to-infrastructure (V2I) communication mechanisms to involve both types of communications in this proposed approach [31].

Machine learning is applied and comprehended to analyze and enhance the system’s working through the proposed methodology. So, relevant to this, in the next section, we first work on the zone-based zone of relevance (ZoR) and zone of forwarding (ZoF) [32,33]. The pre-caching involves placing the content on vehicles like emergency messages, road accident conditions, weather conditions, and traffic congestion as early predictions by the vehicles in the proposed work. The CNN machine learning model is adopted to train and test the model through effective early content prediction for road safety and fewer traffic incidents.

### 3.2. Zone of Relevance (ZoR)

The zone of relevance (ZoR) is based on the vehicle’s velocity and direction of use through its position. The ZoR is constructed to deliver emergency messages in VANET. In the ZoR, continuous messages are exchanged among the vehicles working in the same directory to uphold the exchange of the messages and other relevant technologically advanced features for VANET safety. Time T is set for the periodic messages in ZoR [34]. The expected period for sending the message is [T–1.5T]. This time is random after some delay in the message forwarding and exchanging. ZoR is specially designed to contact through emergency messages through VANET description. The emergency messages are forwarded and exchanged from one to another point to deliver successful event handling in VANET. In our approach, the Geocast-based technique is adopted with ZoR to send the pre-cashed road safety instructions and messages using machine learning. This enhances the early prediction of the road conditions from every other predicted environment. In our approach to the final content prediction for all emergency conditions, the messages or instructions are composed of three main parts; the ID of the vehicle or V2I infrastructure that generated the message, the ZoR specification, and finally, the actual messages created for the final zone-based prediction of messages [35].

### 3.3. Construction of Zones

Zones are constructed based on the clusters mechanism in the proposed VANET architecture. The zone has vehicles that provide similar or heterogeneous properties and resources that combine the collaborative system to enhance the VANET environment for balancing cache content availability and network delay. Let $Z_{i}=\left\{Z_{1},\;Z_{2},\;Z_{3},\;\ldots \ldots .,Z_{n}\;\right\}$, declared as a set of all selected zones inside the network. Every $Z_{i}$ has its range $Z_{size}$ for based on the radius length $RAD_{length}$, the number of vehicles N, and distance from RSU K. Every zone considers two main essential aspects in our approach; one is vehicular nodes and the other is cache content placement. Every node relates to every other node inside the single zone, and every zone relates to every other zone next to it. We construct the zones for the proposed approach using an unsupervised learning algorithm. Additionally, we set interior boundaries for all vehicles through $Interior_{bounday}$, and all interior nodes with the same zone as $Interior_{nodes}$. Equation (1) shows the final zone selection through every zone with the number of entries in the zone table $Z_{table}$.

(1) $$Z_{i}\;\leftarrow \;\frac{1}{\left|Z_{table}\right|}\sum_{p_{zone}\in P_{zone}}N_{n}$$

The proposed zone-based machine learning algorithm was designed. Table 2 is used to select parameters used inside the content placement and selection algorithm. The table consists of the parameter used with descriptions and explanations in the rest of the research article. We design Figure 1 as the proposed framework for content pre-caching and zone construction techniques using a machine learning approach to design our proposed methodology. Equation (2) shows the total zones with every zone in the zone set to promote the correct zone selection.

(2) $$Z_{i}\;\leftarrow \;\left\{Z_{1},\;Z_{2,\;\ldots \ldots ,\;}Z_{n}\right\}$$

In Algorithm 1, the network is distributed into multiple zones. Every zone is selected according to zone radius. The radius length depicts the area covered by the zone. Importantly, the zone is affected through ZoR and ZoF. In each zone, we determined two forms of nodes: one type is interior nodes, and the other is boundary nodes. Interior nodes inside the zone are responsible for communication and message dissemination for the safety of the vehicles in V2V and V2I environments. In other words, these are responsible for Intrazone communication. The main goal is to Geocast communication among the zonal members. The boundary nodes are selected for Interzone communication. The pre-caching strategies are implemented for adequate safety and road accident prevention based on the zone selection. Besides all this information, a zone-based routing table is maintained, storing the routing paths from which the messages are forwarded and received inside each Zone. The time complexity of Algorithm 1 is measured for all zones from $Z_{1}$ to $Z_{n}$. In every zone, the nodes are selected within boundaries. So, the measured time complexity for Algorithm 1 is O(Log $Z_{n\;}$), which is good in terms of zone selection time in the moving environment of VANET. On the other hand, the space complexity for the algorithm is about storing parameters from Table 1, which is O(n).
**Algorithm 1**: Zone Selection**Input**: Parameters from Table 1
 **Output**: $Z_{i}\;\leftarrow \;\left\{Z_{1,\;}\;Z_{2,}Z_{3,}Z_{4,\;}\ldots \ldots ,Z_{n,}\right\}$
 **Steps**
 1. $Z_{size}$ ← R_select($RAD_{length},\;N,\;K$)
 2. **for** j ← 1 to J do
 3.   $Z_{Nodes}$ ←$\;Node_{total}-Interier_{nodes}$
 4.   $Z_{Nodes}$ ←$\;Node_{total}-Interier_{boundary}$
    **while** (meet criteria) **do**
     **for** k ← 1 to N **do**
       
$Z_{table}$ ← { }
      **End for**
      add i ← 1 to i
     
$Z_{i}$ ← $\frac{1}{\left|Z_{table}\right|}\underset{p_{zone}\in P_{zone}}{\sum }N_{n}$
    **End While**
 5.  return $Z_{i}$ ←$\left\{Z_{1},\;Z_{2,\;\ldots \ldots \ldots ,\;}Z_{n}\right\}$
 6. **End for**

### 3.4. Geocasting

Geocasting is one feature used in our proposed methodology for efficient location-based message dissemination or broadcasting in specific zones. Geocast depends on location-based addresses and enhances the functioning of zone-based communication. The central manager maintains geographic location information through roadside units (RSUs) to sustain Geocasting information [35]. The target areas where Geocasting is potential in this research are proclaimed as zones, i.e., (zone of reference) ZoR, and zone of forwarding (RoF). ZoR is certain in Geocast messages. If any of the messages are required to forward or disseminate in any of the Z_i,_ then ZoR is first used to carry the message and needs to be disseminated within a certain ZoR [36].

### 3.5. Role of RSU and Central Manager

Roadside units (RSUs) are the key element in vehicle-to-infrastructure (V2I) communication in VANET. Any information which is exchanged between $Z_{i}\;\left(Zones\right)$ central content manger, RSU to RSU, and RSU to Vehicles are viable through RSU. The RSU links the vehicles, enhancing the node’s working with specific fixed infrastructure [37]. Accessing the Geocasting address of every node working in zones is possible through the RSU. It fixes, continuously collects, and transmits the location information for every vehicular node joining the network. RSU, in our approach, works on radio-type communication. Additional features we extend in our approach include communication for Geocasting network infrastructure. Other responsibilities are to:

- Extend the communication range for zones $Z_{range}^{i}$ and vehicles V.
- Run applications safely.
- Provide internet connectivity to all other OBUs using connected dominated set algorithms [37,38].

On the other hand, the central manager or content manager node Geocasting the pre-caching content to every zonal vehicle through ZoR and ZoF schemes. The manager node uses machine learning to pre-cache the selected zone and vehicle in every zone. The main work of the content manager is as follows.

- Initially, it set the zones $Z_{i}$ and location-based zonal boundaries placement $Z_{boundary}^{L}$ and kept the record in a specified address table.
- Central Manager collects information from the different vehicles about the road conditions. The continuous information provides adequate knowledge and discovery to convey the latest broadcasting network and settings. The proposed technique is effectively overwhelming and intends the VANET message forwarding.
- It periodically checks all network resources through RSU from zones and provides content, message requests, and responses according to their use. Equation (3) elaborates it as.

(3) $$R_{RSU}^{Z_{i}}=Check\left(RSU_{Zone}^{Message}\right)$$

- After getting the emergency messages $M_{enc}$ from the vehicles V, the central manager CM is equipped with the machine learning model, which trains their aspects based on the emergency messages from the RSUs and vehicles. The training period for the machine learning model is operative and provides the knowledge to discover the proper and effective mechanism to enhance the collective management and portability analysis.
- The machine learning ML module continuously handles the training in this approach, and content is processed based on the parameters passed. The prediction accuracy and traffic congestion control are effectively enhanced when machine-learning-based prediction.
- The content is cached on every relevant vehicle using *ZoR* and *RoF*. The event location and consequences are disseminated through safe messages and cached in every zone for future safety predictions. Equation (4) depicts this knowledge.

(4) $$R_{event}\;\leftarrow \;\;V_{ZoR}*\;\sum M_{ZoR}$$

- Additionally, the content manager CM keeps track of every zone $Z_{i}$, vehicle, incident, and cached content. The content pre-caching means the content placement for every vehicle and provides efficient results and location-based successful location areas depicted in Equation (5).

(5) $$C_{pre-cache}^{Vehicle}=\sum_{RSU}^{CM}\left(V_{i},\;C^{cache},\;D,\right)$$

- The machine learning module attached to the content manager CM early predicts the content placement and caching for the vehicles and enhances the working prediction and other related methodologies.

**Figure 4:**
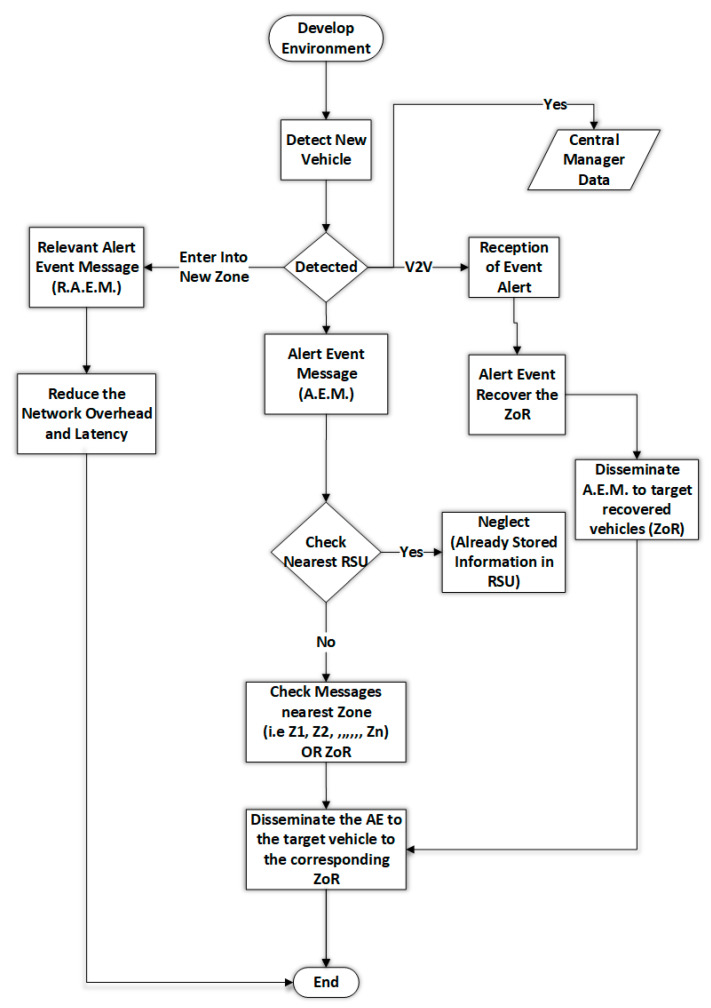
Logical workflow of the proposed methodology.

In Figure 4, we explain the logical workflow of the proposed methodology in the proposed environment where $V_{ZoR}$ join the network. All the pre-registered vehicles that join the network define and provide the relevant details required to set up the technology of the vehicular advance level. After the setup of the VANET environment, the new vehicle $V_{New}^{RoZ}$ is detected and provides the control to add this vehicle through its dedicated $V_{ID}$. The relevant information, such as V, speed, zone, etc., is recorded and saved inside the selected zone, providing the effective zone of relevance. After the detection of the $V_{New}^{RoZ}$ an alert message is issued to all the $V_{ZoR\left(i\right)}$ and data are stored in the central manager $CM_{Geocast}$ for future references and database development. The prediction is based on the Geocast communication defined in Section 2 of the chapter. After the alert, the dissemination is forwarded to the ZoR through the provided emergency messages, and the alert event is recovered by the ZoR. This means the target vehicle successfully finds the relevant $V_{ZoR}$ then disseminates the alert event message to the target vehicles where the final content is required to be pre-cached.

On the other hand, all the targeted vehicles must be detected if the new vehicle enters the zone. An $V_{ZoR}^{Message}$ is forwarded to all the relevant vehicle content and provides effective relevancy in their alert event messages. Early prediction of the vehicles and alert message generation reduce the network latency and overhead of communication and content placement in emergency conditions. In case of emergency, messages are not found, or vehicles are not predicted early, the relevant system will issue the prediction-based accuracy and extend the relevant information with an eviction-based policy. Every $RSU_{ZoR}$ is pre-checked and found to be the best of their system with effective knowledge discovery. The relevant prediction of the nearest zones is handled, providing effective knowledge discovery, and extending the related ZoR. Finally, the messages are disseminated through a pre-caching strategy through effective and forwarded links with relevant ZoR and early prediction for emergency conditions. Algorithm 2 explain the process.
**Algorithm 2:** Data Dissemination Approach**Input:**Table 1, Algorithm 1, $V_{ZoR}$, Geocast Network.
 **Output:**
Target ZoR, Messages Dissimination, Network Development
 **Steps**
 1. $Z_{i}$ ← Algorithm 1
 2. **for**
$K_{i}$ ← 1 to $K_{n}$
**do**
 3.  
$if\;(Detect_{Event}$ ← $V_{ZoR}^{Geocast})$
 4.    $R_{event}$ ←$\;V_{ZoR}*\;\sum M_{ZoR}$
 5.    Disseminate AEM to $\left(\sum_{V}ZoR\right)$
 6.    **else**
 7.   $Select(Z_{i}$ ←$\left\{Z_{1},\;Z_{2,\;\ldots \ldots \ldots ,\;}Z_{n}\right\})$   ∴Algorithm 1
     **end if**
 8.   $send\left(R_{event}\right)\in \left(VANET_{overhead},\;VANET_{latency}\right)$
 9.   **for**
$N_{i}$ ← 1 to $N_{n}$ **do** 10.     $if(RSU_{Zone})$
 11.      $Discard\left(RSU_{Zone}^{Message}==Pre-Stored\right)$
 12.     else
 13.         $Check\left(RSU_{Zone}^{Message}\right)$   $∴\;Z_{i}$ ←$\left\{Z_{1},\;Z_{2,\;\ldots \ldots \ldots ,\;}Z_{n}\right\}$
 14.     $Dissiminate\left(Message_{Zone}^{V}\right)$   ∴ ZoR
 15.   **End for**
 16.  return $Message{\left(s\right)}_{Dissimination}\;(Z_{i}$ ←$\left\{Z_{1},\;Z_{2,\;\ldots \ldots \ldots ,\;}Z_{n}\right\})\;$
 17. **End for**

### 3.6. Machine Learning-Based Content Pre-Caching Predictions

Machine learning-based content caching or pre-caching enhances the popularity of content retrieval and request. ML-based techniques efficiently enhance early content placement based on vehicle location and cache size. There are also context-aware adopting techniques proposed based on ML-based approaches. ML-based content pre-caching improves the QoS of the proposed approach. The content is cached based on V2V and V2I content placement strategies. We pre-cache the content using an ML algorithm for road and deliver safety to cache emergency messages. The proposed technique predicts early content placement to a higher probability of securing safety from road incidents and accidents. Algorithm 3 uses the machine learning model to train and test content pre-caching strategies. Let $ZOR_{i}$ be the zone of relevance for content caching. $MN_{M}^{store}$ is the content on the central manager node. $V_{i\to j}^{0}\;and\;P_{i\to j}^{0}$ are the initial velocity and position of the vehicle used in the performance network. $ML_{Model}^{pre-cached}$ is the proposed machine learning-based pre-caching model for the proposed method.

The model is trained based on parameters like vehicle delivery content towards other vehicles based on previous content requests, road conditions, vehicle running speed, and moving paths to other vehicles or RSU. The model is trained based on provided emergency messages dataset. The training prediction shows effective results. The training provides every node with dataset values and collaborative techniques to flow the proposed methodology. The central manager node is updated about the new content pre-caching model to pre-cache content. The pre-caching enhances the content predictions and enhances results for vehicle node transmission. The model is trained on all the data through pre-caching. The idea of pre-caching is the advanced prediction of any road incidents and road accidents to save human lives and resources. To improve the system’s efficiency, extensive training data and time for training are required to achieve the desired accuracy of the system.

The dataset description is provided in the IV(A) section. Algorithm 3 uses a machine learning-based content pre-caching approach based on input and output parameters. Initially, we use data preset to select the zone and vehicle where we pre-cache the content. The dataset is retrieved from the real environment and other vehicular nodes to predict the selected model. The positions and velocity of the selected vehicle are finalized in the preset data stage. Once the dataset is prepared according to network configuration and requirements, we train the proposed model using machine learning in the next step. The system is trained based on provided dataset. Values are predicted based on every measured value to finalize the position-trained model. The steps are registered after training the selected CNN-based ML model. Now it’s time to test the resultant model with the testing dataset. The predictions are captured with accuracy, average delay, cache hit ratio, and network throughput. The results are obtained from the predicted values and dataset range. Algorithm 3 is a machine learning algorithm using the CNN model. The measured computational time complexity of the CNN model in Algorithm 3 is O (k, n, d^2^ with and space complexity is O(n), where n defines the total dataset used in testing and training).

The main steps in Algorithm 3 are

1. Get zone from Algorithm 1;
2. ZoR for dissemination from Algorithm 2;
3. The originated data are captured and handled through the manager nodes, with road and traffic conditions captured through every incident vehicle;
4. Store data in the manager node from the real environment;
5. Set initial velocity and position to the initial stage;
6. Set the training data for valuable variables;
7. Calculate the prediction value for every sample dataset;
8. Calculate the error;
9. If step 8 is not good, then training starts for the provided dataset;
10. The model is effectively trained.

**Algorithm 3:** Machine Learning-Based Content Pre-Caching**Input**: Table 1, Algorithm 1, $V_{ZoR}$, $Geocast\;Network,\;Data_{train}$ **Output**: Target ZoR, Messages Dissimination, Network Development **Steps**: **Prepare Dataset**
1. $Z_{i}$← Algorithm 1      ∴Get Zone from Algorithm 1
2. $ZOR_{i}$← Algorithm 2      ∴ZoR for dissemination from Algorithm 2
3. $Data_{VANET}\;\leftarrow \;\left(\begin{array}{c} Video,\;Emergy\;Road\;Conditions,\;traffice\;\\ conditions,\;Control\;messages \end{array}\right)$         ∴The originated data are captured and handled through the Manager         nodes with road and traffic conditions captured throughout every         incident vehicle.
4. $MN_{M}^{store}\;\leftarrow step\;3$       ∴Store data in the manager node from the real environment
5. $\mathrm{Initialize}:\;V_{i\to j}^{0}\;\&\;P_{i\to j}^{0}$   ∴Set initial velocity and position to the initial stage
**Train Model**
6. **for**$\;i=1\;to\;Data_{train}$
**do**   ∴Set the training data for valuable variables
7.  **for** j=1 to N
**do**      ∴Calculate the prediction value for every sample dataset
8.   $calculate\_error\left(prediction\;Vlaues\right)$    ∴Calculate the error
9.    $if\left(!step\;8\right)$
10.   $Apply\left(weighted\;values\right);$
11.     $ML_{Vanet\;}\;\leftarrow Trained\left(\right)$
12.   **End for**
13.  **End for**
14. **Return**: $ML_{Model}^{pre-cached}\left(Trained\right)$
**Test Model**
15. Prepare: $Data_{Testing}^{pre-cache}$
16. **for** k=1 to M
**do**
17.   $Test\left(Data_{Testing}^{pre-cache}\right)\;\leftarrow Prediction-Results;$
18.   $Capture\left(\begin{array}{c} prediction\;accuracy,\;\\ average\;delay,\;hit\;ratio,\;throughput \end{array}\right);$
19. **End for**
20. **Return**: Graphical Results


A dedicated CNN-based machine learning model is adopted in our proposed system. The machine learning model is embedded in RSU. This makes the efficiency of the proposed system more accurate early prediction of content pre-caching. The machine learning scenarios are easily met using dependent machine learning objectives. The proposed system was trained through plenty of previous pre-caching examples from different vehicle requests. The algorithm efficiently predicts the content request based on previous request rates of vehicles.

Consequently, the proposed machine learning-based content pre-caching strategy provides full convenience to people who drive on highways. This research focused on improving the zone-based content pre-caching through machine learning. The proposed machine learning pre-caching provides the network with a new pre-caching protocol to enhance the safety of passengers and drivers. The main goal of the research is to provide an effective and reliable safety control system. ZoR is considered to enhance the services of the system with enlist effectiveness. This paper focused on emergency pre-caching, which enhances vehicle security events in ZoR. To achieve a pre-caching, a CNN-based supervised machine learning algorithm is used. After evaluating the proposed system and discussing its working, we explained performance evaluation with obtained results from the proposed technique.

## 4. Results

**Figure 5:**
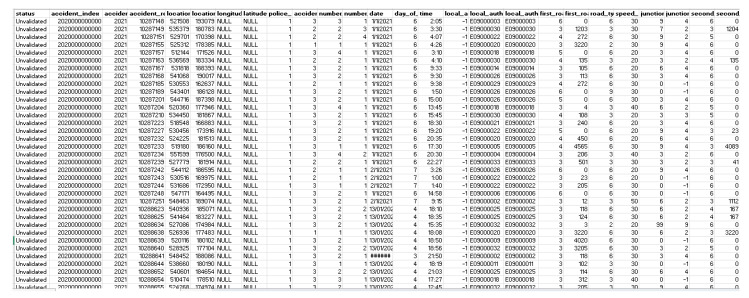
Dataset description with sample values used inside the research.

We implement our proposed technique in NS-2 to evaluate the proposed model. The simulation environment setup contains the simulation experiments performed on VANET and machine learning supportive computers. Figure 5 explains the environment setup and variables description. The VANET network is designed to achieve the final and practical behavior of proposed VANET devices. Four parameters are selected to investigate the performance of the proposed system. The proposed algorithms and methodologies are discussed and addressed in the previous version of the proposed work. The results are evaluated by comparing the results with zone-based content pre-caching [39] and Geo-SID [40], and the throughput parameter is implemented to control the network congestion. Table 3 shows simulation parameters with values.

### 4.1. Dataset Description

The supervised machine learning technique required labeled datasets for training and testing purposes. The numerical results are based on a real dataset that demonstrates the effectiveness of the proposed content pre-caching scheme. We are working on a novel technique to solve the problem of congestion handling and load balancing. The dataset is obtained and generated using the VANET environment in NS-2. The main characteristics of the dataset are indicated using Figure 5 with sample values. There are 115,585 records of the dataset.

Furthermore, the proposed model is based on the CNN model for content pre-caching for emergency messages. We have used pre-existing datasets and performed some cleansing work to improve the dataset’s quality to get more accurate results. We split our dataset into two resultant techniques, the trained dataset, and the second testing dataset. The results section clearly defines the dataset with the description of a result. Experiments are performed on every dataset to analyze the performance of the proposed system. The simulation experiments are performed on VANET and machine learning-supportive computers. The model testing is performed on PyCharm over Python and sublime text 3. A 96.3% accuracy was achieved by the model after 70 training iterations. The machine learning-based results presented in this section are related to training and testing datasets for the cleaned dataset. The convolution neural network is based on the trained model with testing features. After training, the novel model has achieved 96.3% accuracy and provides effective and enhanced network operations over CNN.

### 4.2. Machine Learning Model Evaluation

**Figure 6:**
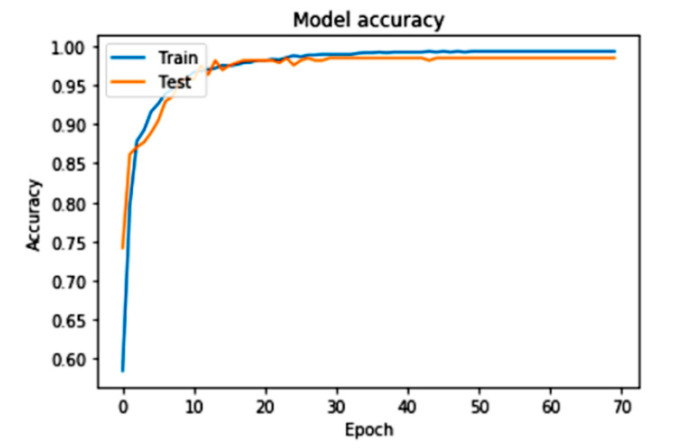
Accuracy of the proposed machine learning CNN model.

We train our model for every class of attribute from the dataset to evaluate the performance of the proposed system. Testing is performed on every class with classification and model performance analysis tools. The result for each attribute is shown in Figure 6 as a correlation map. Figure 6 shows the accuracy of the model for training the model. The training shows that the system’s training prediction is effectively enhanced and provides practical training comparisons on every dataset.

(6) $$\mathrm{Accuracy}\;=\frac{\mathrm{Number}\;\mathrm{of}\;\mathrm{Classified}\;\mathrm{Samples}}{\mathrm{Total}\;\mathrm{Samples}}\;$$

**Figure 7:**
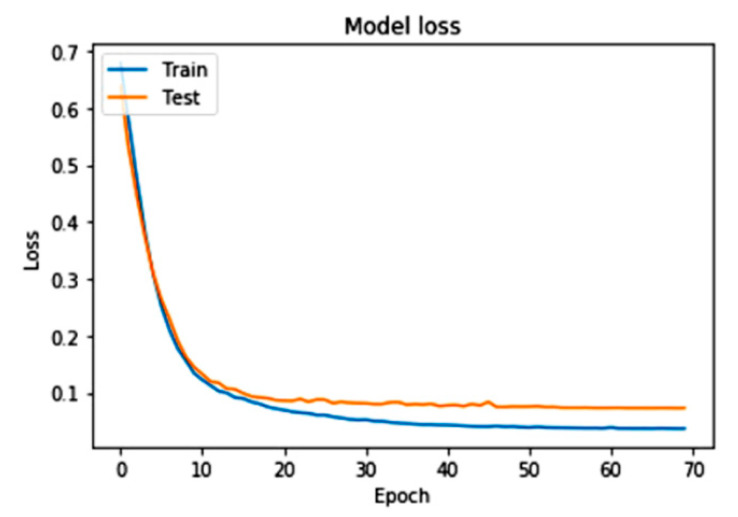
Proposed CNN model loss with train over the testing dataset.

Figure 6 shows that the model’s accuracy increases rapidly with the final predicted dataset range. We performed maximum iterations (epoch) on a model from the dataset to train the required model. We captured the loss of the proposed system over the requested dataset. According to the loss of the model, the system performance is measured with a training and test dataset. Figure 7 shows the very minimum loss of the proposed model. The training and testing loss in the model is according to the predefined dataset to train and test the proposed model with iterations.

**Figure 8:**
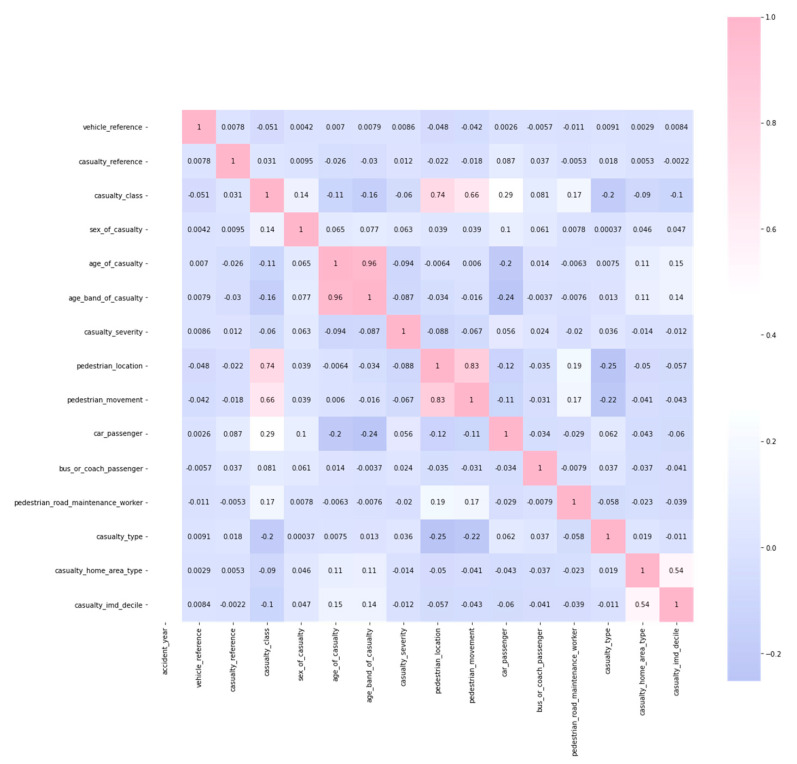
Correlation map.

After preparing the model overtraining and testing, we evaluate the system performance through a confusion matrix. The metric effectively adopted the number classes on the dataset and performed the analysis system. Figure 8 shows the correlation map with the accuracy of the values to promote the system performance with analysis techniques. According to the accuracy of the performance measurement we evaluated, we reached 96.3% accuracy for the selected number of classes from the dataset to get promoted.

### 4.3. Cache Hit Ratio

**Figure 9:**
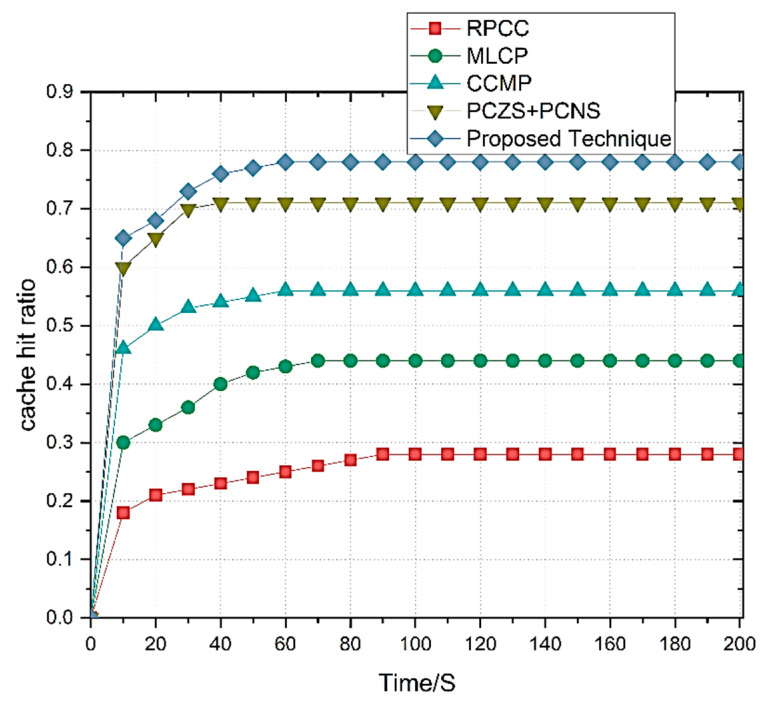
Cache hit ratio of the proposed technique.

The cache hit ratio in this system accommodates the cache requests from the cached vehicles. RSU cache hit ratio also compares the number of requests received from vehicles from respective Zones. ZoR is applied for the cache hit ratio. We have selected 50 vehicles to track for a cache hit ratio compared to the proposed techniques. The graph in Figure 9 shows the adaptability of different techniques adopted through the dynamic nature of the curves, including the main curve. The cache hit ratio of the RPCC, MLCP, and CCMP shows satisfactory results related to the other techniques. We established the review process and defined the effective results based on these techniques.

On the other hand, the zone-based content pre-caching, i.e., PCZS+PCNS, shows results that received higher cache rates than other techniques. PCZS+PCNS selects the pre-caching zones based on content popularity. The proposed technique shows better results than PCZS+PCNS because its machine learning-based early prediction module effectively pre-caches the content based on accuracy in the prediction. The proposed technique effectively chooses the content pre-caching nodes based on the ZoR-based data dissemination approach, extending the working and providing accurate prediction results. Thus, the proposed technique improves the cache hit ratio with higher prediction accuracy.

### 4.4. Prediction Accuracy

**Figure 10:**
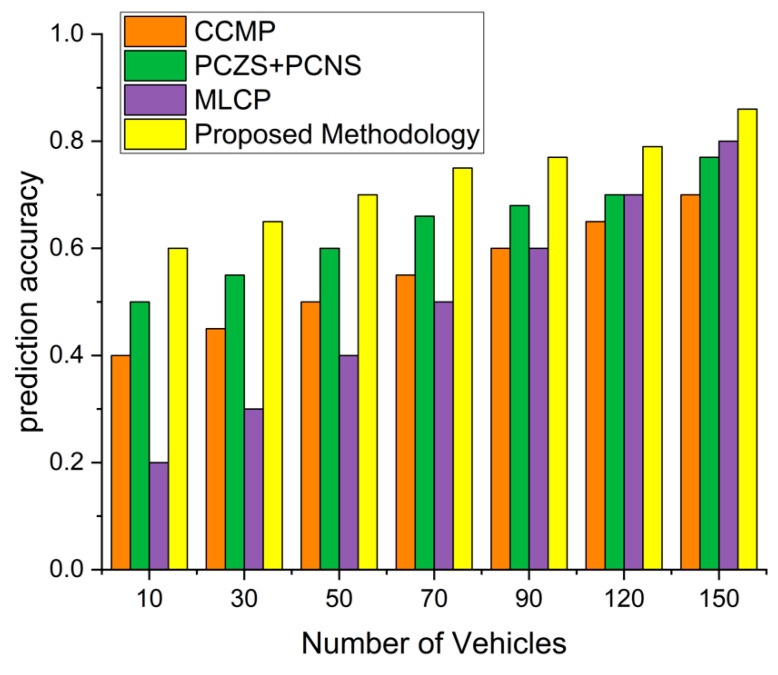
Pre-caching Prediction Accuracy of the proposed technique.

In Figure 10, the proposed methodology shows the highest prediction accuracy compared to the CCMP, MLCP, and PCZS+PCNS. In the figure, the MLCP technique follows the ML-based traffic estimation, neglecting the vehicular motion detection and dynamic characteristics of the pre-caching strategies to predict the knowledge and accuracy-based prediction mechanism. On the other hand, the CCMP has more prediction accuracy, and PCZS+PCNS obtained more effective and predictive results than other techniques. These techniques follow the regions which estimate the predictive values and provide the highest accuracy of predicted results. However, the proposed content pre-caching techniques for emergency and road traffic conditions using ZoR-based zones for ML predictions effectively result in 17% more accurate results. We encounter road topology, congestion, traffic, speed estimation, ZoR, and event alert-based techniques to estimate and provide higher accuracy results than previous techniques.

The ML-based early prediction enhances traffic accuracy and provides more early-based prediction not to affect the prediction results and enhance the traffic flow in the network. With an increasing number of nodes, the prediction accuracy and highest impact metrics increase and estimate the prediction results by comparing and providing effective estimation techniques. The traffic flow changes and enhancements of the traffic incidents follow the road and incident conditions accurately and effectively. The proposed techniques’ early prediction policy effectively utilizes the effectiveness literature on the proposed techniques in traffic jams and emergency and road accident conditions. It enhances the predictions to solve the actual values for better and more effective results comparisons.

### 4.5. Average Delay

**Figure 11:**
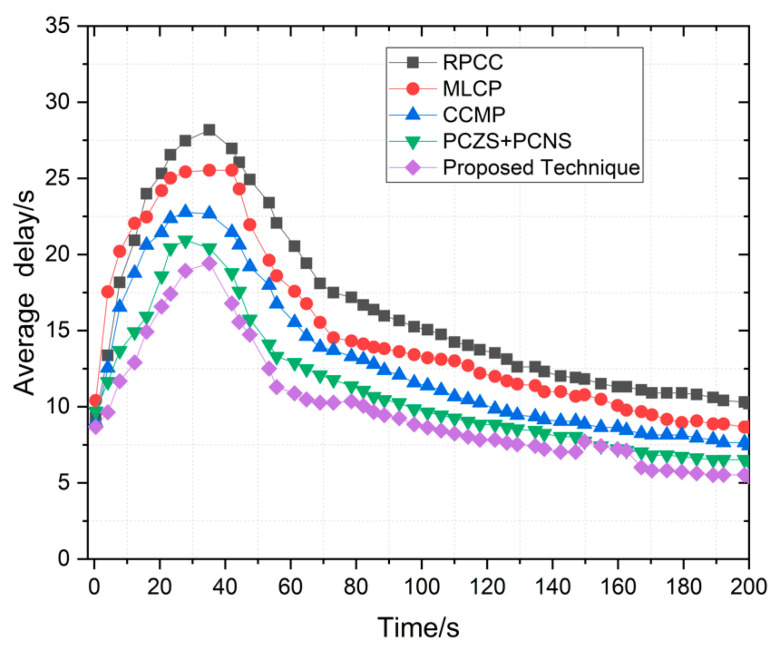
Average Delay of proposed technique compared with RPSS, MLCP, CCMP, PCZS+PCNS.

**Figure 12:**
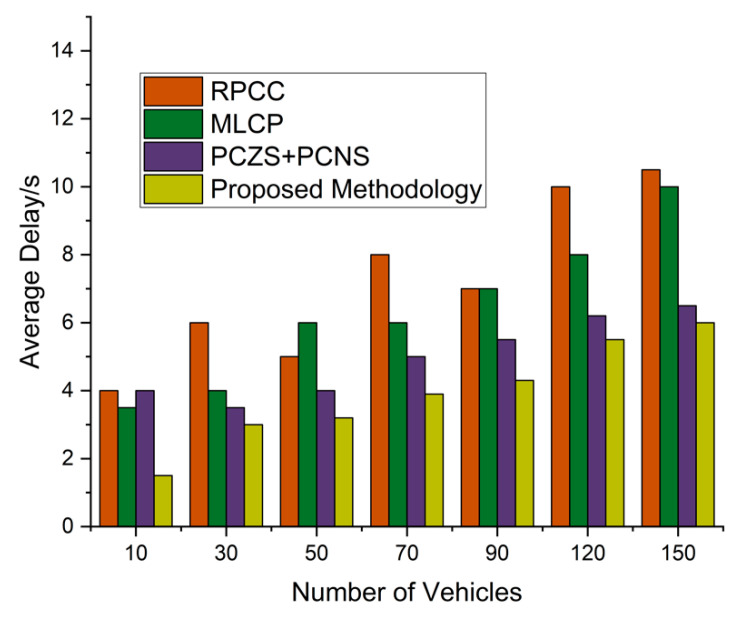
Average delay on the number of nodes for the proposed methodology.

The average delay is the waiting time to receive data requests from any respective zone or network. We tracked the average delay time by adopting 50 and 150 vehicles in this simulation results. We check our proposed system based on these two main parameters to evaluate the results. The graph in Figure 11 shows the results explained easily to find the final results from the proposed methodology. The trends rise at the initial stages, then fill for the average delay to summarize the results. The results explain the end methodology and the results to simulate effective management. Initially, the cached content on the different zone for content is null; with the simulation time, the simulation ends with an effective proposed methodology. Over time RPCC, MLCP, CCMP, and PCZS+PCNS compared the results with the proposed technique. These results effectively enhance the working functionality of the proposed technique. Figure 12 shows that network latencies with different degrees effectively reduce and enhance effective collaborative results.

The existing techniques provide a lack of effective results of the proposed technique. The updating mechanism is compared with the compared approach. The dynamic adaptability from the network analysis shows that it provides results comparisons as earlier peaks and with the decline with time. The proposed technique provides less average delay than the existing techniques in dynamic adaptability.

They are adding more vehicles—up to 150—with the network’s average delay of the RPCC, MLCP, and PCZS+PCNS. The average delay increases when the number of vehicles is continuously added to the existing network. Road traffic and network traffic increased by adding more vehicles, and under this condition, our proposed system performs well and shows minimum average delay compared to existing techniques. The number of vehicles increased the prediction accuracy to provide the content pre-caching to enhance the operational performance of the proposed system. The prediction process contains the network traffic latency and shows the minimum content information regarding other vehicles’ traffic flow. The pre-caching is evolved by developing the proposed mechanism to enhance and provide the pre-caching accuracy values.

### 4.6. Network Throughput

**Figure 13:**
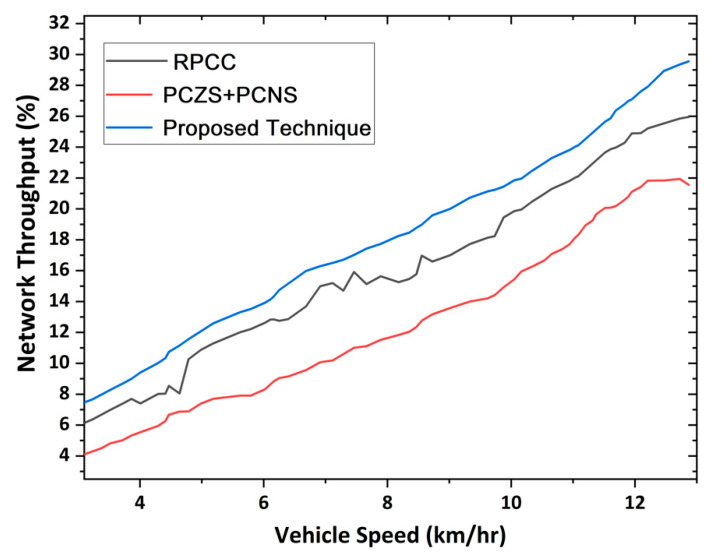
Network throughput for congestion control in the proposed approach.

The network shows cooperative communication among vehicles, infrastructure, and RSUs. Network throughput shows the successful content pre-caching under lower delay in VANET. The performance of the proposed network infrastructure is explained using network throughput. Maximizing the network throughput explains less network congestion using the proposed technique. The cache hit ratio improved in Section 4.3, effectively showing the utilization of the network caching, which overcomes the burden of vehicles to search for the content which causes network congestion. Once the vehicles join the zone using algorithm 1, it utilizes the pre-cached content easily using Algorithm 2, which enhances the network throughput, ultimately reducing congestion inside the network. The proposed protocol reduces the data request collision probability by setting up the required features. The network uses all the protocols defined inside the network with the ability to participate in overcoming network congestion. Figure 13 shows the network throughput of the proposed technique. The correlation between vehicle speed and throughput is when the vehicle speed grows.

Then the cache hit ratio and services delivery performance (i.e., throughput) impact the network’s overall performance as speed matters in highway scenarios where the pre-caching is a significant challenge resolved in this problem and a high value of throughput is achieved. Network throughput explains the average ratio of the average delay, pre-caching and prediction accuracy, and cache hit ratio when vehicles were moving along the highway. Figure 4 shows the throughput with network performance, like average delay, pre-caching, accuracy, and cache hit ratio. According to the results in Figure 4, our proposed technique matches the vehicle speed. In response, the RPCC and PCZN+PCNS techniques only show results when the vehicle speed is slower. Under high speed, their throughput goes down. According to the results, it is clear that when the vehicle moves at high speed, the throughput of these techniques is compromised. The results also show that the throughput of accessing the content caching and network congestion control enhances with time. The increase in the throughput is the availability of content data and the running network smoothly.

### 4.7. Statistical Results

**Figure 14:**
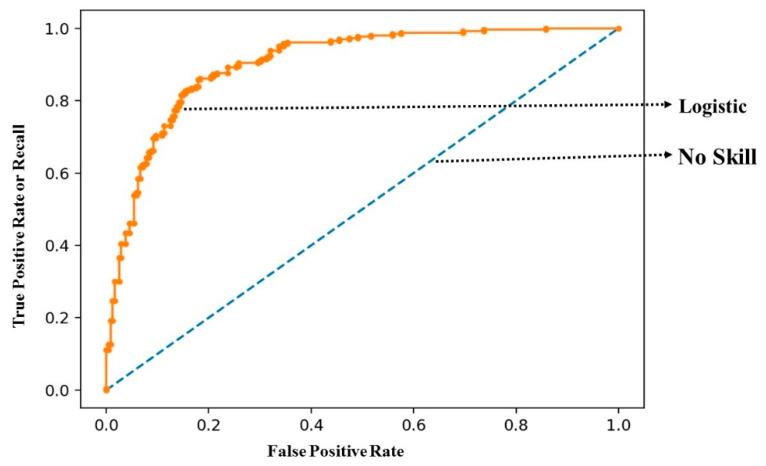
ROC curve to show the tradeoff between specificity and sensitivity values for the proposed method.

The initial statistical results were performed using a ROC (receiver operating characteristic) curve. The ROC shows the diagnostic ability of the machine learning classifier used in the method. Figure 14 shows the ROC curve to show statistical analysis. The results of ROC show that the curve is closer to the top left corner, which shows better results. The closer the curve comes to the 45-degree diagonal of the ROC space, the less accurate the test is.

**Figure 15:**
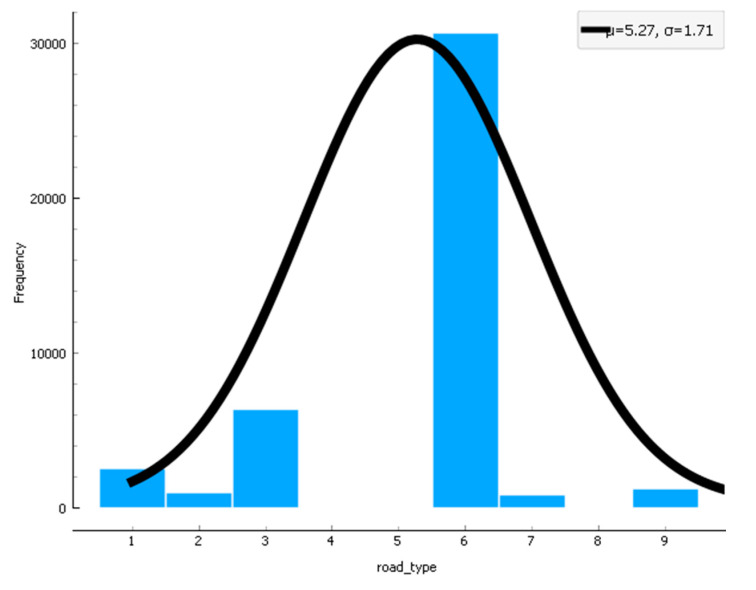
Confidence interval for road type used in the scenario.

**Figure 16:**
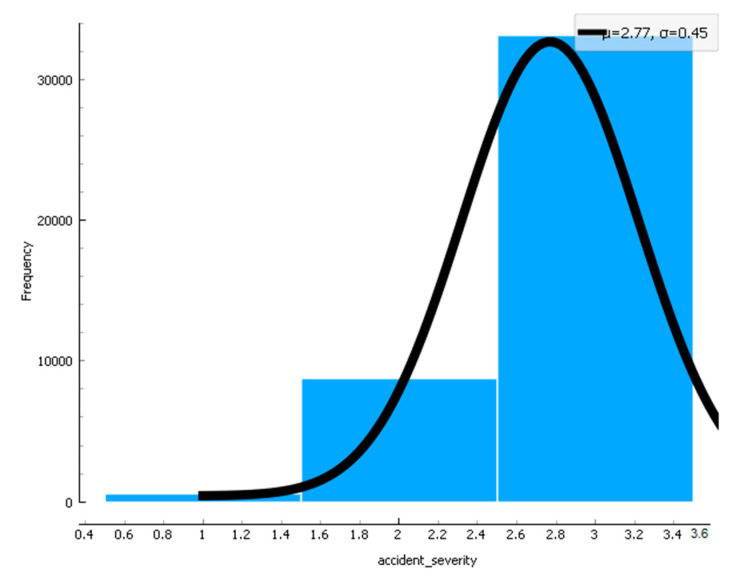
Confidence interval for accident severity is used in the scenario.

**Figure 17:**
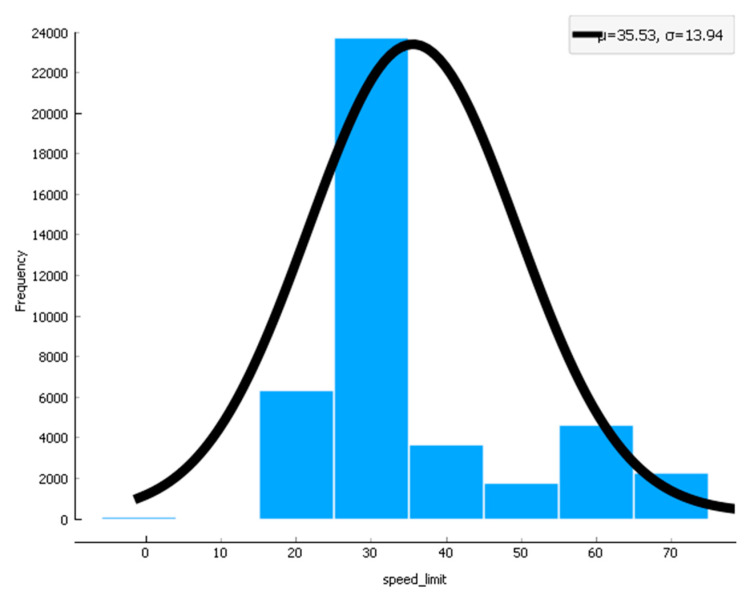
Confidence interval for the speed limit used in the scenario.

In Figure 15, Figure 16 and Figure 17, the confidence intervals of the road type used in the research data, accident severity, and speed limit for the provided dataset are used. The results in Figure 15 show that when road type changes, the cached data distribution also has. Likewise, in Figure 16, the accident severity increases when the congestion is not controlled under the situation and scenario. Similarly, in Figure 17, the curve shows that when speed limits reduce, the data distribution enhances, and when speed limits increase, the data distribution reduces in the pre-caching scenario.

## 5. Conclusions and Future Directions

The research concluded that zone construction and selection, content dissemination, and machine learning-based content pre-caching resolve the problem of pre-caching for early decision-making and results from prediction. In this research, we encounter the limitations faced by the VANET. A simulation-based model is introduced for the proposed work’s preliminary introduction, enhancing the correlation for the said objectives. We work on a vehicular ad hoc network to propose a zone-based content pre-caching policy. First, we zone with selected parameters described in Section 3 for selecting the zone and its zonal head for content pre-caching. Zonal coverage and their access scope are set for selecting the zone. Second, after selecting the zones and zonal heads, we encounter the content dissemination approach to special condition safety and special conditions messages towards every zonal head node. Finally, we adopt the machine learning model to train for content pre-caching based on the zones, zonal heads, high availability, maximum utilization, and others to place and predict the content for the VANET. The results are evaluated for the proposed technique through NS-3 with efficient and reliable content placements and content delivery ratio. The reliability and adaptability of the network are evaluated through the proposed simulation-based technique. The outperform are evaluations for cache hit ratio, hop count, cache prediction accuracy, and high adaptability of content in each zone.

In the future, further improvements are focused on the following. The pre-caching approach is extended to adopt the deep learning-based prediction of pre-caching content to improve the results further. A geographical-based machine learning model can be introduced to pre-cache the content based on location privacy. Moreover, security aspects can also be implanted after including the results recommendations. Hijacking the caching node can bring a serious security breach inside the network. Through this, the entire network can also be compromised.

## Figures and Tables

**Table 1:** Common limitations of the related works from the literature.

S. No.	Author	Methodology	Parameters	Contributions	Limitations
1	Guo et al. [16]	The pre-caching node selection algorithm	prediction accuracy and average delay	Effectively pre-cache the content	Overhead involved when loading the content to the node
2	Fourati et al. [17]	Name-based content caching	Network delay and performance	Handle the content normal caching	It does not work on cache prediction accuracy
3	Alioua et al. [18]	Stackelberg Game Approach	Congestion and content quantity	Overcome network congestion	Does not work on the network throughout, and network performance metrics
4	Liu et al. [19]	Two-layer distributed content caching	Caching capacity, transmission rates	Performance analysis	Congestion handling, throughput, and network performance
5	Wang et al. [20]	Game-based caching incentive scheme	Content caching	Enhance content caching results	The cost of caching is very high
6	Xue et al. [21]	Hidden Markov model-based content pre-caching	Delivery ratio, hop count, and network latency values, ML technique to enhance the working performance	Enhance the caching scheme for the efficiency of the proposed technique	ML technique for the efficiency of the content caching technique
7	Pereira et. al. [22]	RSU caching and vehicular pre-caching	Content pre-caching, delivery time, load balancing	Caching performance, delivery time	It does not consider cache prediction accuracy, average delay, or cache hit ratio
8	Yao et al. [23]	Integrated networking approach	Deep reinforcement learning approach	Content caching and system enhancements	No discussion about congestion handling and network performance
9	Zhang et al. [25]	Joint optimization scheme of content caching	ML-based content caching, joint-implemented strategy, policy for efficiency	Clusters or zones which are not effective	There is no use of clusters or zones or throughput
10	Hu et al. [27]	Mobility-aware edge caching approach	Handling through network performance and measurement	Large-scale mobility aware cost estimation model	Does not find content pre-caching strategies and every time post content caching policies

**Table 2:** Parameters with the description used for the proposed methodology.

S. No.	Parameters	Definition	Description
1	$Z_{i}$	Zone declared in VANET, i.e., Z1 for zone 1, Z2 for zone 2, and so on	Every zone defines its communication and provides effective feedback for zone-based content caching. There are several zones concerning the number of vehicles and RSUs.
2	$CM_{y}^{D}$	Central manager for vehicles data storage	The central manager is a controller server node responsible for communication and effective results computation and enhancements methodology.
3	$CM_{y}^{dec}$	Central manager decides the communication	The communication is started and decided by the central communication manager with effective and collaborative work and feature requirements. The central manager communicates and works effectively with other nodes.
4	RSU	Roadside unit to process the request and communication devices	Roadside units are responsible for communicating with the central manager and vehicles. These are fixed stations on the end of the highways to provide content and other control facilities to the VANET environment.
5	CT	Content storage in popularity order (ascending or descending order)	The content is stored with the popularity order of the network resources, enhancing the access and privileges mechanism to control and communicate among these challenges.
6	$C_{zi}\;$	Congestion of zone	The congestion in every zone is balanced through the proposed techniques. The congestion is related to the content and requests for content placement and enhancements.
7	CH	Cluster heads for zones	Every zone holds a selected cluster head, the cluster’s part, and makes effective cluster collaborations to discuss and provide the cluster-based implementation policies. The discussed results provide effective zone-based content pre-caching and enhancement strategies.
8	$X_{red}\;$	Zone radius	Every zone holds a radius that consumes the zone area and provides the system enhancement features to collect and discuss the resulting privileges.
9	ML	Machine learning algorithms for vehicles	We adopted a machine learning vehicles-based approach to handle the content congestion and enhanced the features for the collaborative workings.
10	Content cache	Content caching for passing from machine learning	The content caching is passed through machine learning approaches and provides the collaborative work methodology for content cache placement.
11	$V2V_{com}$	Communication points from vehicle-to-vehicle	The communication point from the vehicular addresses provides the collective methodology for the results and discussions to effectively elaborate and provide the working methodologies.
12	$V2I_{com}\;$	Communication points from vehicle to infrastructure	The vehicle infrastructure contains an appropriate communication point that shows the collection of infrastructure movements and provides enhancement strategies for infrastructure-based content placement schemes.
13	$R_{r}$	Requested content of size r	The content requested by the consumer node is of size r, where r is the threshold value set in the algorithm to provide effective caching placement and strategies implementations.
14	$D_{r}$	Delivered content of size r	The content which is delivered to the requested vehicles is of size r.
15	$Req_{data}$	Requested content with data requested	This shows the data and content request from the requested node toward the content placement and enhancement strategies.
16	$DM_{Node}^{Z}$	Data rate among manager nodes and vehicles	The data rate for the manager node and vehicles shows the data delivered among these nodes for proper implementation.
17	$Z_{table}$	Intrazone table	This table shows the communication among different zones and their coordination among them.

**Table 3:** Simulation parameters.

S. No.	Parameters	Used Values
1	Popularity Model	Zipf
2	Framework	VANET environment in NS-2
3	Simulation Area	300 × 300
4	Wireless Connectivity	Wi-Fi
5	Publisher/RSUs	10
6	Subscribers/Vehicles	150
7	Cache Size	200
8	File Size	Chunks on Demand Basis
9	Message Transformation	15–18 Sec
10	Mobility Model	2D-Random Directions
11	Number of Simulations Run	200
12	Simulation Time	The 1200 s
13	Road Conditions	One Way Highway
14	Broadcast Interval Time	The 50 s
